# High levels of soluble RAGE are associated with a greater risk of mortality in COVID-19 patients treated with dexamethasone

**DOI:** 10.1186/s12931-022-02220-5

**Published:** 2022-11-05

**Authors:** Lee Butcher, Jun-Cezar Zaldua, Jose A. Carnicero, Karl Hawkins, Janet Whitley, Rangaswamy Mothukuri, Phillip A. Evans, Keith Morris, Suresh Pillai, Jorge D. Erusalimsky

**Affiliations:** 1 The Cellular Senescence and Pathophysiology Group, Cardiff Metropolitan University, Llandaff Campus, Western Avenue, Cardiff, CF5 2YB UK; 2 Welsh Centre for Emergency Medicine Research, Emergency Department, Morriston Hospital, Swansea Bay University Health Board, Swansea, SA6 6NL UK; 3 Fundación para la Investigación Biomédica del Hospital Universitario de Getafe, Getafe, Spain; 4 Medical School, Swansea University, Swansea, UK

**Keywords:** Mortality, Prognostic, Biomarkers, sRAGE, IL-6, NEWS2, Dexamethasone, COVID-19, SARS-CoV-2

## Abstract

Blood levels of the soluble receptor for advanced glycation end-products (sRAGE) are acutely elevated during the host inflammatory response to infection and predict mortality in COVID-19. However, the prognostic performance of this biomarker in the context of treatments to reduce inflammation is unclear. In this study we investigated the association between sRAGE and mortality in dexamethasone-treated COVID-19 patients. We studied 89 SARS-CoV-2 positive subjects and 22 controls attending the emergency department of a University Teaching Hospital during the second wave of COVID-19 and measured sRAGE at admission. In positive individuals sRAGE increased with disease severity and correlated with the National Early Warning Score 2 (Pearson’s r = 0.56, p < 0.001). Fourteen out of 72 patients treated with dexamethasone died during 28 days of follow-up. Survival rates were significantly lower in patients with high sRAGE (> 3532 pg/mL) than in those with low sRAGE (p = 0.01). Higher sRAGE levels were associated with an increased risk of death after adjustment for relevant covariates. In contrast, IL-6 did not predict mortality in these patients. These results demonstrate that sRAGE remains an independent predictor of mortality among COVID-19 patients treated with dexamethasone. Determination of sRAGE could be useful for the clinical management of this patient population.

**Supplementary Information:**

The online version contains supplementary material available at 10.1186/s12931-022-02220-5.

## Background

The receptor for advanced glycation end-products (RAGE) and its soluble forms have been increasingly implicated in innate immunity and inflammation [1, 2]. RAGE is a transmembrane receptor that binds a variety of ligands, including advanced glycation-end products, cell adhesion proteins and molecules originating from damaged mammalian cells and pathogens [3, 4], transducing intracellular signals which lead to the activation of pro-inflammatory processes. In healthy adults RAGE is constitutively expressed at high levels in the lungs and skin, whereas in cardiometabolic and inflammatory diseases it is up-regulated in different cell types across the organism [1]. Similarly, expression also increases during the host response to infection [2]. Soluble RAGE (sRAGE) originates largely from the proteolytic cleavage of the extracellular portion of membrane-bound RAGE, a process which is upregulated by inflammatory signals [5]. Previous studies have shown that elevated sRAGE levels predict mortality in acute lung injury [6], acute respiratory distress syndrome [7] and sepsis [8]. Consistent with those findings, several studies have recently reported that very high sRAGE levels were associated with COVID-19 severity and/or mortality [9–12].

The RECOVERY trial recently identified the corticosteroid dexamethasone as the first drug to significantly improve survival in COVID-19 patients [13]. Corticosteroids modulate the host response to infection and thus could potentially affect recognised relationships between inflammatory biomarkers and outcomes. In this respect, the prognostic performance of sRAGE in the context of treatments to reduce inflammation has not been previously investigated. Accordingly, the objective of the present study was to examine the association between sRAGE and mortality in dexamethasone-treated COVID-19 patients.

## Methods

Study design and patient selection: 120 patients admitted with suspected COVID-19 through the Emergency Department and the Respiratory Assessment Unit of a University Teaching Hospital in South Wales, UK, were enrolled in a prospective observational study from September 2020 to February 2021 during the second wave of the pandemic. Written informed consent was obtained from the patient or a legal representative during the admission process. Patients aged less than 18 years, those receiving anticoagulant treatment and those who declined to participate were excluded. All individuals were tested for SARS-CoV-2 using real-time reverse transcriptase polymerase chain reaction from a nasopharyngeal swab. Individuals testing negative were included in the control group. Patients received treatment in a general COVID-19 ward or in an intensive care unit (ICU) in accordance with the prevailing guidelines at the time of the study. Where appropriate, dexamethasone was given intravenously at a dose of 6 mg/day for up to 10 days as described in the RECOVERY trial [13]. Time to death was measured for up to 28 days from admission. Eight patients for whom a blood sample for biomarker evaluation was not available were retrospectively excluded. An additional patient whose sRAGE was 10,640 pg/mL had a National early warning score 2 (NEWS2) of 15 (falling > 1.5 times above the interquartile range); this was deemed a NEWS2 leverage point and hence this patient was also excluded, leaving 111 remaining participants for analysis. The study was approved by the South-West Wales Research Ethics Committee (Wales REC 6).

IL-6, CRP and sRAGE were measured in serum prepared from blood samples taken within 24 h after admission. In dexamethasone-treated patients, blood was drawn within an average time frame of 10 h before and 17 h after the start of dexamethasone treatment. Serum was stored at − 80 °C until the time of analysis. Measurements were carried out in duplicates using the following commercially available sandwich ELISAS: human IL-6 DuoSet ELISA (DY206), human CRP DuoSet ELISA (DY1707) and human RAGE Quantikine Immunoassay (DRG00), all from R&D Systems (Abingdon, UK), as described by the manufacturer. The sRAGE immunoassay detects both cleaved sRAGE and esRAGE. The origin of the samples was blinded to the operator. Intra-assay coefficients of variation were < 5.5% in all cases. There were no significant differences in the average levels of biomarkers between samples taken before or after dexamethasone administration.

Details on other baseline parameters and statistical analyses can be found in the Additional file 1.

## Results

**Fig. 1:**
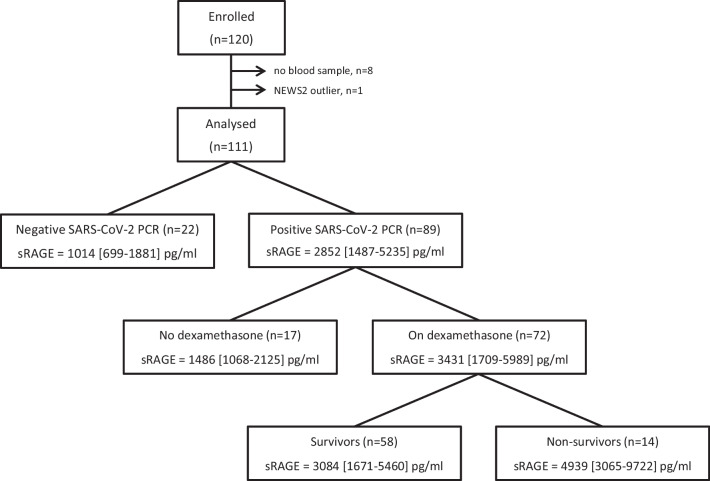
Diagram depicting the composition of participants enrolled in the study

**Fig. 2:**
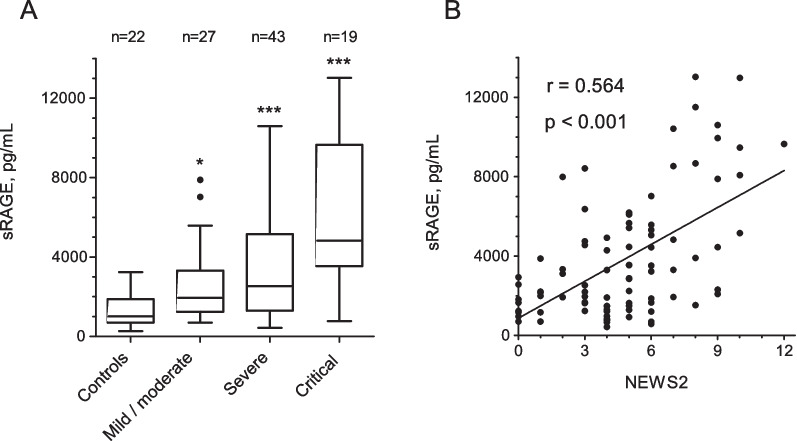
Association between sRAGE levels and COVID-19 severity: **A** Box and whiskers plot (Tukey method) comparing sRAGE levels between non-COVID-19 controls and COVID-19 patients with increasing disease severity; * p < 0.05, *** p < 0.001 by Kruskal–Wallis with Dunn’s multiple comparisons test vs control. **B** Pearson’s correlation between sRAGE levels and NEWS2 scores in COVID-19 patients

A diagram depicting the composition of the 111 participants analysed in the study is shown in Fig. 1. Eighty-nine subjects were SARS-CoV-2 positive and 22 were negative. On admission, median sRAGE levels were significantly higher in SARS-CoV-2 positive subjects than in those that were negative (2852 [1487–5235] pg/mL vs 1014 [699–1881] pg/mL, p < 0.001). sRAGE levels increased with COVID-19 severity (p < 0.001) (Fig. 2A) and showed a positive correlation with NEWS2 (Fig. 2B), but not with IL-6 (see Additional file 2: Table S1). Furthermore, when NEWS2 was binarized using a clinically relevant threshold, sRAGE was found to be significantly higher in individuals with NEWS2 ≥ 5 (n = 47) compared to those with NEWS2 < 5 (n = 42) (4450 [2091–7889] pg/mL vs 1862 [1171–3160] pg/mL, p < 0.001).

Of the 89 SARS-CoV-2 positive subjects, 72 subjects were treated with dexamethasone. The baseline demographic and clinical characteristics of dexamethasone-treated and non-treated patients are summarized in Table 1. Dexamethasone-treated patients had significantly higher levels of sRAGE, CRP and IL-6, as well as a non-significant tendency to show a slightly reduced kidney function. In addition, these patients were more likely to have a history of hypertension compared to their non-dexamethasone counterparts but did not show a significant difference in the prevalence of other co-morbidities. They also had a higher NEWS2 on admission, they spent more days in hospital, and a larger proportion of them were also treated with Remdesivir.

**Table 1:** Baseline characteristics of COVID-19 patients by dexamethasone treatment

Variable	All		No dexamethasone		Dexamethasone		P**
	n		n		n		
Age in years, median [IQR]	89	63.0 [53.0–72.0]	17	56.0 [46.5–69.5]	72	63.0 [54.3–72.8]	0.128
Male, %	89	41.6	17	29.4	72	44.4	0.258
Smoking history, %	75	46.7	15	46.7	60	46.7	1.000
BMI, median [IQR], kg/m^2^	55	30.3 [26.6–34.5]	13	26.6 [23.0–32.7]	42	31.2 [28.4–35.4]	0.057
sRAGE, median [IQR], pg/mL	89	2852 [1487–5235]	17	1486 [1068–2125]	72	3431 [1709–5989]	***0.002***
CRP, median [IQR], pg/mL	78	72.0 [35.3–156.0]	15	14.0 [5.0–49.0]	63	90.0 [47.0–202.0]	** < *0.001***
IL-6, median [IQR], pg/mL	89	21.8 [7.9–55.3]	17	9.5 [4.4–24.7]	72	23.7 [8.7–60.5]	***0.025***
DDimer, median [IQR], ng/mL	85	816 [512–1644]	15	678 [567–1119]	70	895 [501–1722]	0.223
eGFR, median [IQR], mL/min/1.73m^2^	84	69.5 [60.0–88.5]	17	78.0 [68.0–110.0]	67	68.0 [59.0–86.0]	0.060
Diabetes, %	89	22.5	17	11.8	72	25.0	0.240
Hypercholesterolemia, %	89	5.9	17	5.9	72	5.6	0.985
Hypertension, %	89	29.2	17	5.9	72	34.7	***0.019***
Cardiovascular disease*, %	89	10.1	17	17.6	72	8.3	0.252
DVT or PE, %	89	3.4	17	0.0	72	4.2	0.392
COPD, %	89	10.1	17	5.9	72	11.1	0.520
Cancer, %	89	12.4	17	5.9	72	13.9	0.367
NEWS2, median [IQR]	89	5.0 [3.0–6.0]	17	3.0 [0.0–5.0]	72	5.0 [3.3–7.0]	***0.004***
Remdesivir, %	89	37.1	17	0.0	72	45.8	** < *0.001***
Admitted to ICU, %	89	9.0	17	0.0	72	11.1	0.150
Days in hospital, median [IQR]	89	7.0 [3.0–13.0]	17	0.0 [0.0–9.0]	72	7.5 [4.0–13.8]	***0.001***

n depicts the number of participants for whom baseline information for the corresponding variable was available

*BMI* body mass index, *COPD* chronic obstructive pulmonary disease, *CRP* C reactive protein, *DVT* deep vein thrombosis, *eGFR* estimated glomerular filtration rate, *ICU* intensive care unit, *IL-6* interleukin 6, *NEWS2* national early warning score 2, *PE* pulmonary embolism, *sRAGE* soluble receptor for advanced glycation-end products

^*^Comprises coronary heart disease, heart failure and/or stroke. **Statistically significant results are highlighted in bold italics

During the 28 days follow-up period 14 dexamethasone-treated patients died, whereas no deaths were registered in the non-dexamethasone group. Patients who died had significantly higher levels of sRAGE than those who survived (median [IQR]: 4939 [3065–9722] vs 3084 [1671–5460] pg/mL, p = 0.042). A univariate Cox proportional hazards regression analysis of the entire dexamethasone-treated group demonstrated that sRAGE (entered as a natural logarithm-transformed continuous variable) was a significant predictor of mortality (HR = 2.18, 95%CI 1.03–4.60, p = 0.041). Several other parameters were also associated with mortality in this group, including age, a NEWS2 score ≥ 5 on admission, a diagnosis of COPD and cancer. Of note, IL-6 did not predict mortality in dexamethasone-treated patients (see Additional file 2: Table S2).

**Fig. 3:**
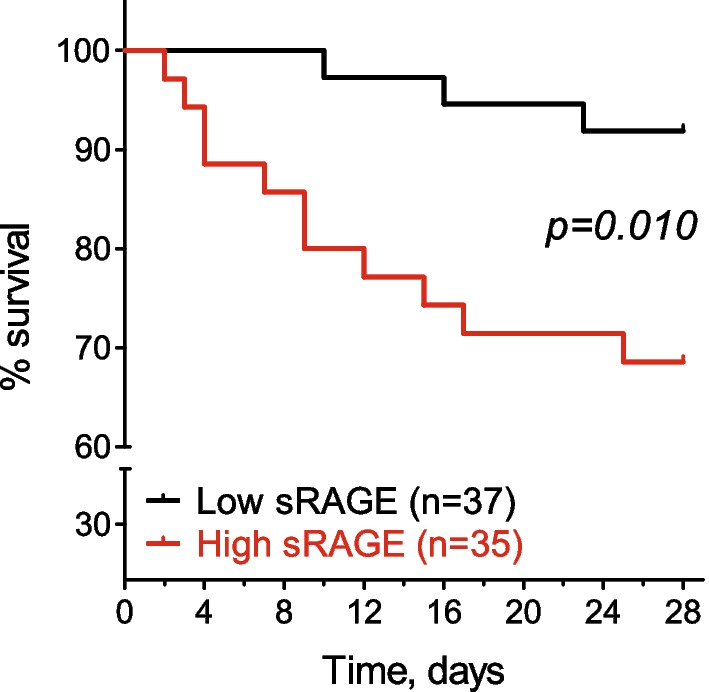
Kaplan–Meier survival curves of dexamethasone-treated COVID-19 patients with low and high levels of sRAGE. sRAGE was categorized into low and high levels according to a cut-off value of 3532 pg/mL as described in the main text

In the absence of a clinically relevant sRAGE threshold to discriminate patients at a higher risk of death, we dichotomised this parameter based on the estimation of Youden’s index. This value corresponded to the 52nd percentile of the sRAGE distribution, namely 3532 pg/mL. Kaplan–Meier survival curves for dexamethasone-treated patients categorised according to this threshold, showed a significant difference in survival rates (p = 0.01), with 93% of patients with low sRAGE being alive at the end of the follow-up compared to 68% of those with high sRAGE (Fig. 3).

To examine further the association between sRAGE and mortality in dexamethasone-treated COVID-19 patients we compared four multivariate regression models adjusting for those variables that predicted mortality in the univariate analysis. Table 2 shows that sRAGE remained a significant predictor of mortality with little or no attenuation across all the adjusted models; the behaviour of the models was similar when sRAGE was entered as continuous or binary variable. Altogether, the results indicate that in dexamethasone-treated patients the risk of death increases over two-fold with every unit increase in Ln-sRAGE and more than four-fold when sRAGE is dichotomised using the threshold value of 3532 pg/mL.

**Table 2:** Multivariate Cox proportional hazards regression analysis of the relationship between sRAGE and mortality in dexamethasone-treated patients

	Ln sRAGE			high sRAGE*		
	HR	95% CI	*P*	HR	95% CI	*P*
Unadjusted	2.18	1.03–4.60	0.041	4.57	1.27–16.40	0.020
Model 1	2.39	1.10–5.22	0.028	4.76	1.32–17.16	0.017
Model 2	2.31	1.10–4.85	0.026	4.54	1.22–16.94	0.024
Model 3	2.54	1.21–5.32	0.014	6.32	1.59–25.07	0.009
Model 4	2.16	1.01–4.61	0.046	4.25	1.11–16.25	0.035

Model 1: multivariate model adjusted for age

Model 2: model 1 additionally adjusted for COPD

Model 3: model 2 additionally adjusted for cancer

Model 4: model 2 additionally adjusted for NEWS2 ≥ 5

^*^sRAGE > 3532 pg/mL

Integrated area under the ROC curve analysis was used to compare the performances of sRAGE and NEWS2 in their ability to predict mortality (see Additional file 2: Table S3). This showed that while NEWS2 performed better than sRAGE, the combination of sRAGE and NEWS2 increased the predictive accuracy of the model by 0.6%. Importantly, no multi-collinearity between sRAGE and NEWS2 was detected.

## Discussion

In this study we demonstrate that among hospitalized COVID-19 patients treated with dexamethasone, high levels of sRAGE at the time of admission predict mortality over the next 28 days. Importantly, we found that this relationship was independent of other predictors, including age, COPD, cancer or the NEWS2 score. Furthermore, we show that when comparing between patients with sRAGE levels below and above 3532 pg/mL, the survival rates of the later are ~ 25% lower. Thus, this study provides new evidence relating high levels of sRAGE with looming mortality, highlighting the potential relevance of using this biomarker for risk stratification of COVID-19 patients treated with dexamethasone.

The present results are consistent with findings from recent investigations showing that sRAGE is considerably raised in hospitalised COVID-19 positive patients, predicting adverse outcomes [9–12]. However, in contrast to our work, those investigations did not take account for the use of corticosteroids. Indeed, some of the earlier sRAGE studies had started before dexamethasone was widely adopted following the formal demonstration that it reduced mortality in patients requiring supplemental oxygen [13]. On the other hand, while other studies reported similar prognostic associations for various classic markers of inflammation, including IL-6, TNF-α and CRP [11, 14, 15], our findings suggest that IL-6 does not predict mortality in dexamethasone-treated patients. Thus, our results extend previous findings, indicating that sRAGE could be a better biomarker to evaluate mortality risk in hospitalized COVID-19 patients if they are receiving dexamethasone therapy.

A clinical sRAGE threshold for the identification of patients at risk of poor outcomes in acute respiratory conditions is yet to be established. Our findings show that in COVID-19, a threshold set at 3532 pg/ml is suitable for mortality risk prediction of dexamethasone-treated patients. This value was 3.5-fold higher than the median value of sRAGE in our COVID-19 negative controls, the latter being well within the range of sRAGE concentrations reported for normal individuals in many other studies [5]. In comparison, in a cohort of COVID-19 pneumonia patients of whom a minority (< 10%) had been treated with dexamethasone, Lim et al.[12] derived a threshold of 5833 pg/mL. Whether these differences reflect variations in disease severity between the two patient populations or in some other characteristic, remains speculative. Nevertheless, further studies will be required to either validate or modify these values.

Although our results are based on observational data, they provide insights into the biological mechanisms underlying the relationship between sRAGE and mortality in COVID-19. sRAGE has been connected to pathways of both inflammation and lung epithelial cell injury [5, 6, 16]. In this respect, the fact that sRAGE continues to predict mortality in dexamethasone treated-patients while IL-6 does not, suggests that extensive alveolar epithelial damage is the primary driving force behind the increase in sRAGE. In making this distinction, it should be noted that although sRAGE can act as an extracellular decoy of membrane-bound RAGE when administered to animal models, thereby playing an anti-inflammatory role [17], endogenous sRAGE levels, i.e. those generated in vivo, may not be sufficient to neutralize pro-inflammatory RAGE ligands [18]. Alternatively, raised sRAGE levels may simply reflect the outcome of a signal amplification mechanism whereby ligand binding to surface RAGE induces further RAGE expression and also upregulates the RAGE-cleaving metalloproteases MMP9 and ADAM10, thus resulting in an increased sRAGE shedding into the circulation [5]. In line with this possibility, increasing evidence shows that a subgroup of endogenous RAGE ligands, collectively known as damage-associated molecular patterns (DAMPs), which are secreted from damaged or dead cells, including lung cells, are elevated in severe COVID-19 patients [4]. Furthermore, the alveolar epithelium is known to be the tissue with the highest constitutive RAGE expression [4] and high blood levels of MMP9 have been recently detected in hospitalised COVID-19 patients [19, 20]. Thus, RAGE overstimulation by DAMPs in the lungs, may lead to an acute increase in sRAGE formation, reflecting the underlying extensive cellular injury, and the imminent irreversible organ damage and death.

The NEWS2 early warning score is used in UK National Health Service hospitals for detection and response to clinical deterioration in adult patients. In agreement with recent reports, we also found that a NEWS2 score ≥ 5 predicted mortality in this condition [21, 22]. Furthermore, we found that NEWS2 scores correlated with sRAGE and that the later improved the predictive accuracy of NEWS2. One could argue that the increase in the accuracy of the prediction model was relatively small. However, the fact that sRAGE remained a significant predictor of mortality after adjustment for NEWS2, indicates that this biomarker is clinically relevant, thus suggesting that the combination of the two variables could represent a better tool to help with the selection of patients who might benefit from additional interventions to prevent further deterioration.

Strengths of this study include its prospective design and the availability of blood samples for biomarker analysis despite the overwhelming hospital conditions during the critical phases of the pandemic. Nonetheless, the study also has several limitations. This was a single centre study and might not represent the wider population. Participants were mostly white Europeans, so findings might not generalise to other ethnicities, particularly given the fact that sRAGE is known to vary with race. A further limitation of this study is that the observed associations could have been influenced by factors not included in the multivariable models. For example, because there were missing data on renal function, we could not evaluate the potential confounding effect of this variable. In addition, the study considered dexamethasone-treated patients as a whole and hence the extent to which the results apply in the same measure to patients receiving different forms of oxygen support remains to be established. Finally, sRAGE levels in COVID-19 patients have been reported to be at their highest upon hospital admission, decreasing gradually over the next 7 days [9]. In our study, we could not collect blood samples at various time points and therefore, we could not ascertain if changes in sRAGE levels might be useful as potential indicators of resistance to dexamethasone treatment.

In summary, despite the above-mentioned limitations, our study adds to the growing literature relating acute rises in sRAGE to respiratory infections and might have implications for understanding the biological pathways and treatments which influence outcomes in these conditions. Future studies should aim to establish if lowering sRAGE levels might be a treatment goal to improve survival rates in COVID-19.

## Supplementary Information

**Additional file 1.** Supplementary methods.

**Additional file 2.** **Table S1.** Relationships between sRAGE, NEWS2 and IL-6. **Table S2.** Univariate analysis for potential predictors of mortality in dexamethasone-treated COVID-19 patients. **Table S3.** Performance of sRAGE and NEWS2 in their ability to predict mortality in dexamethasone-treated COVID-19 patients

## Data Availability

The datasets used and/or analysed during the current study are available from the corresponding author on reasonable request.

## Abbreviations

COPD: Chronic obstructive pulmonary disease
CRP: C reactive protein
DAMPs: Damage-associated molecular patterns
ICU: Intensive care unit
IL-6: Interleukin 6
NEWS2: National early warning score 2
RAGE: Receptor for advanced glycation end-products
sRAGE: Soluble receptor for advanced glycation-end products
