# Supplementary material for: High levels of soluble RAGE are associated with a greater risk of mortality in COVID-19 patients treated with dexamethasone
Source: Respir Res. 2022 Nov 5;23:303. doi: 10.1186/s12931-022-02220-5 (PMC9637291; doi:10.1186/s12931-022-02220-5)
Supplement: Supplementary file 1 — Additional file 1. Supplementary methods. [file 12931_2022_2220_MOESM1_ESM.docx]

**Supplementary Methods**

**Baseline parameters**

Baseline clinical and demographic data were extracted from the hospital records. COVID-19 severity was defined as being either mild, moderate, severe or critical. Mild cases included patients that were discharged within 24 h of admission. Moderate cases included individuals whose hospital stay was between 1 and 5 days. Severe cases consisted of individuals who spent more than 5 days in hospital, whereas critical cases included individuals who were either admitted to ICU or who died in hospital. National early warning score 2 (NEWS2) was calculated on arrival to hospital as previously described [1]. NEWS2 was dichotomised using a score of 5 as the cut-off. Body Mass Index (BMI) was calculated as weight in kilograms divided by height in meters squared. All routine biochemical parameters were determined by standard clinical chemistry methods. The estimated glomerular filtration rate (eGFR) was calculated using the 2021 CKD-EPI creatinine equation [2].

**Statistical analysis**

Continuous variables are reported as medians with IQR. Categorical variables are presented as percentages. Differences in baseline characteristics between dexamethasone-treated and non-treated patients were compared by Mann-Whitney test for continuous variables and by χ2 test for categorical variables. Pearson’s correlation was used to determine the relationship between two continuous variables.

The relationship between sRAGE and mortality was assessed by Cox proportional hazard regression analysis using SSPS v.23 for Windows (SPSS Inc., Chicago, IL, USA). In this analysis sRAGE and IL-6 values were natural logarithm transformed given their skewed distribution. We also categorized sRAGE using Youden’s index. A univariate analysis was initially performed to identify those variables that conferred a statistically significant risk of death. Variables with missing data were excluded to maximize the statistical power. Multivariate models were adjusted for those variables which predicted mortality in the univariate model. Given the relatively small number of events, it was considered inappropriate to include all the relevant covariates in one model. Accordingly, four successive models were constructed. Model 1 was adjusted for age; model 2 was additionally adjusted for chronic obstructive pulmonary disease (COPD); model 3 included the variables in model 2 plus cancer; and model 4 included the variables in model 2 plus NEWS2 ≥5. The prognostic performances of sRAGE and NEWS2 were evaluated by computing the integrated area under the ROC curve (IAUC) for every day over the time interval between 15 and 28 days.

Kaplan-Meier analysis was performed with sRAGE dichotomized into high and low levels based on a threshold estimated from Youden’s Index. The time to event was calculated as the period between baseline blood sampling and the date of death or being censored after 28 days. Survival curves were compared by the Mantel-Cox log-rank test using Prism v.5.01 (GraphPad Software Inc., La Jolla, CA, USA).

**References**

1. Royal College of Physicians. National Early Warning Score (NEWS) 2: Standardising the assessment of acute-illness severity in the NHS. Updated report of a working party. London: RCP, 2017. www.rcplondon.ac.uk/news2 [Accessed 20 July 2022].

2. Inker LA, Eneanya ND, Coresh J, Tighiouart H, Wang D, Sang Y, Crews DC, Doria A, Estrella MM, Froissart M, et al: New Creatinine- and Cystatin C-Based Equations to Estimate GFR without Race. *N Engl J Med* 2021, 385:1737-1749.
