# Supplementary material for: High levels of soluble RAGE are associated with a greater risk of mortality in COVID-19 patients treated with dexamethasone
Source: Respir Res. 2022 Nov 5;23:303. doi: 10.1186/s12931-022-02220-5 (PMC9637291; doi:10.1186/s12931-022-02220-5)
Supplement: Supplementary file 2 — Additional file 2. Table S1. Relationships between sRAGE, NEWS2 and IL-6. Table S2. Univariate analysis for potential predictors of mortality in dexamethasone-treated COVID-19 patients. Table S3. Performance of sRAGE and NEWS2 in their ability to predict mortality in dexamethasone-treated COVID-19 patients [file 12931_2022_2220_MOESM2_ESM.docx]

**Table S1:** Relationships between sRAGE, NEWS2 and IL-6

| Variable | sRAGE | NEWS2 | IL-6 |
| --- | --- | --- | --- |
| sRAGE | - | 0.564 ^***^ | 0.128 |
| NEWS2 | 0.564 ^***^ | - | 0.198 |
| IL-6 | 0.128 | 0.198 | - |

Values correspond to the Pearson correlation coefficients (r) between two variables; *** p<0.001

Abbreviations: IL-6, interleukin 6; NEWS2, national early warning score 2; sRAGE, soluble receptor for advanced glycation-end products

**Table S2**: Univariate analysis for potential predictors of mortality in dexamethasone-treated COVID-19 patients (n=72; deaths=14)

| Variable | HR | 95% CI | *P* |
| --- | --- | --- | --- |
| Age | 1.09 | 1.04-1.14 | ***0.001*** |
| Male gender | 1.12 | 0.39-3.24 | 0.830 |
| Ln sRAGE | 2.18 | 1.03-4.60 | ***0.041*** |
| Ln IL-6 | 1.23 | 0.83-1.81 | 0.298 |
| Diabetes | 0.76 | 0.21-2.72 | 0.670 |
| Hypercholesterolemia | 1.46 | 0.19-11.20 | 0.713 |
| Hypertension | 1.50 | 0.52-4.33 | 0.453 |
| Cardiovascular disease * | 3.42 | 0.95-12.34 | 0.060 |
| DVT and PE | 1.74 | 0.23-13.33 | 0.593 |
| COPD | 9.30 | 3.15-27.44 | ***< 0.001*** |
| Cancer | 3.94 | 1.32-11.78 | ***0.014*** |
| NEWS2 ≥5 | 10.91 | 1.43-83.41 | ***0.021*** |
| Remdesivir | 0.83 | 0.29-2.39 | 0.731 |
| Admitted to ICU | 2.10 | 0.58-7.51 | 0.256 |
| Days in hospital | 0.99 | 0.96-1.03 | 0.747 |

* Comprises coronary heart disease, heart failure and/or stroke

Abbreviations: COPD, chronic obstructive pulmonary disease; DVT, deep vein thrombosis; ICU, intensive care unit; IL-6, interleukin 6; NEWS2, national early warning score 2; PE, pulmonary embolism; sRAGE, soluble receptor for advanced glycation-end products

**Table S3**: Performance of sRAGE and NEWS2 in their ability to predict mortality in dexamethasone-treated COVID-19 patients

|  | IAUC | 95% CI |
| --- | --- | --- |
| Ln sRAGE | 0.683 | 0.672-0.693 |
| NEWS2 | 0.815 | 0.807-0.823 |
| Ln sRAGE + NEWS2 | 0.821 | 0.810-0.831 |

Abbreviations: IAUC, integrated area under the ROC curve; IL-6, interleukin 6; NEWS2, national early warning score 2; sRAGE, soluble receptor for advanced glycation-end products
